# Simultaneous Determination of Over-the-Counter Pain Relievers in Commercial Pharmaceutical Products Utilizing Multivariate Curve Resolution-Alternating Least Squares (MCR-ALS) Multivariate Calibration Model

**DOI:** 10.1155/2019/1863910

**Published:** 2019-07-31

**Authors:** Heba Shaaban, Ahmed Mostafa, Zahra Almatar, Reem Alsheef, Safia Alrubh

**Affiliations:** Department of Pharmaceutical Chemistry, College of Clinical Pharmacy, Imam Abdulrahman Bin Faisal University, King Faisal Road, P.O. Box 1982, Dammam 31441, Saudi Arabia

## Abstract

The quality of over-the-counter (OTC) pain relievers is important to ensure the safety of the marketed products in order to maintain the overall health care of patients. In this study, the multivariate curve resolution-alternating least squares (MCR-ALS) chemometric method was developed and validated for the resolution and quantification of the most commonly consumed OTC pain relievers (acetaminophen, acetylsalicylic acid, ibuprofen, naproxen, and caffeine) in commercial drug formulations. The analytical performance of the developed chemometric methods such as root mean square error of prediction, bias, standard error of prediction, relative error of prediction, and coefficients of determination was calculated for the developed model. The obtained results are linear with concentration in the range of 0.5–7 *μ*g/mL for acetaminophen and 0.5–3.5 and 0.5–3 *μ*g/mL for naproxen and caffeine, respectively, while the linearity ranges for acetyl salicylic acid and ibuprofen were 1–15 *μ*g/mL. High values of coefficients of determination ≥0.9995 reflected high predictive ability of the developed model. Good recoveries ranging from 98.0% to 99.7% were obtained for all analytes with relative standard deviations (RSDs) not higher than 1.62%. The optimized method was successfully applied for the analysis of the studied drugs either in their single or coformulated pharmaceutical products without any separation step. The optimized method was also compared with a reported HPLC method using paired *t*-test and *F*-ratio at 95% confidence level, and the results showed no significant difference regarding accuracy and precision. The developed method is eco-friendly, simple, fast, and amenable for routine analysis. It could be used as a cost-effective alternative to chromatographic techniques for the analysis of the studied drugs in commercial formulations.

## 1. Introduction

Pain relievers are among the most widely consumed medications worldwide. These medications are used for relieving inflammatory pain conditions [1]. Pain relievers such as acetaminophen, ibuprofen, naproxen, aspirin, and caffeine are available as over-the counter (OTC) pharmaceutical preparations.

Ibuprofen is a nonsteroidal anti-inflammatory (NSAID) drug. Its chemical name is 2-(4-isobutylphenyl)-propionic acid. Ibuprofen is commonly used for treating pain and inflammation in musculoskeletal disorders, especially in rheumatoid arthritis [2]. Paracetamol (acetaminophen) is an antipyretic drug that acts by inhibiting the synthesis of prostaglandins. Aspirin (acetylsalicylic acid) is a NSAID drug used for relieving pain, fever, and inflammation [3]. However, caffeine has no analgesic activity of its own, and it is widely used in combination with aspirin and acetaminophen as it has adjunct action for the enhancement of relieving pain. Incorporating caffeine in OTC pain relief formulations increases their activity by ∼40% because of the ability of caffeine to facilitate the absorption of other drugs and to cause constriction of the cerebral blood vessels [3]. Naproxen is also a NSAID drug that acts by inhibition of prostaglandin synthesis [1].

Because of the growing consumption of OTC pain relievers, controlling the quality of these medications is highly required in order to ensure their efficiency. Therefore, developing new reliable, fast, and cost-effective methods for analyzing such drugs is of paramount importance.

Implementation of green procedures in analytical laboratories is of paramount interest in order to minimize the negative environmental impacts [4, 5]. Green analytical chemistry aims at replacing nongreen analytical methodologies with green alternatives that use and generate no/less hazardous chemicals [6, 7]. Compared to chromatographic methods that utilize sophisticated instruments and consume or generate large volumes of organic solvents, spectrophotometric methods are faster, more economical, and greener, making them fully functional alternatives. However, spectrophotometric techniques suffer from lack of selectivity especially in case of severe spectral overlap and matrix interferences, the selectivity of such techniques can be enhanced by using multivariate calibration models [8, 9]. The development of multivariate calibration methods such as multivariate curve resolution-alternating least squares (MCR-ALS) (based on signals mathematical resolution) enables the resolution and quantification of strongly overlapped spectra of multicomponent samples [10]. MCR-ALS is characterized by the ease of implementation and its ability to overcome bands overlapping. MCR-ALS has recently attracted attention in the analytical chemistry society and has been shown to provide enhanced resolution and improved quantitative determination of different analytes in samples of increasing complexity such as environmental matrices [11] and pharmaceutical and agricultural samples [10, 12]. The main advantage of using MCR-ALS is the possible recovery of the spectral information of target analytes and unknown interferences [9]. Different analytical methods have been developed for the individual assay of the OTC pain relievers and also for some of their combinations including HPLC (e.g. [13–15]) and spectrophotometry (e.g., [16–20]).

To the best of our knowledge, no studies have been reported for simultaneous determination of paracetamol, aspirin, caffeine, naproxen, and ibuprofen in pharmaceutical formulations using multivariate calibration methods. The aim of this work is to develop a green, fast, low-cost analytical method utilizing MCR-ALS multivariate calibration for simultaneous determination of the cited OTC pain relievers in their single and coformulated products without any separation step with the aid of easily accessible instruments (e.g., UV spectrophotometer). The proposed method is economic, fast, eco-friendly, and amenable for routine analysis.

## 2. Experimental

### 2.1. Instrumentation and Software

A UV-1800 Shimadzu double-beam spectrophotometer (Shimadzu, Kyoto, Japan) with 1 cm quartz cell was used. The scanning speed was maintained at 2800 nm·min^−1^, and the wavelength range was set from 200 to 400 nm with a bandwidth of 1 nm. Spectra were automatically obtained by Shimadzu UV-Probe 2.62 software. For performing MCR-ALS calculations, MCR-ALS GUI 2.0 software was used with Matlab 2015a [21]. The MCR-ALS algorithm is available at http://www.mcrals.info.

### 2.2. Chemicals and Reagents

The standards used in this study including paracetamol (PAR), acetylsalicylic acid (ASA), ibuprofen (IPF), naproxen (NPX), and caffeine (CAF) were purchased from Sigma-Aldrich (Steinheim, Germany) and certified to contain ≥98%. Methanol (CAS No. 67-56-1) was obtained from Merck (Darmstadt, Germany). Ultrapure water (18.2 MΩ) purified using the PURELAB Ultra water system (ELGA, High Wycombe, UK) was used for sample preparation.

### 2.3. Stock and Working Standard Solutions

Stock solutions of the studied drugs were prepared separately by dissolving 10 mg of each drug in 10 ml methanol to obtain a concentration of 1 mg·mL^−1^. All solutions were kept in dark at 4°C. Working standard solutions were freshly prepared by appropriate dilution in ultrapure water.

### 2.4. Construction of Calibration and Validation Sets

The calibration model (a set of twenty-five calibration solutions) was built using a five-factor, five-level design [22] to cover the linearity ranges of 0.5–7 *μ*g/mL for PAR, 0.5–3.5 *μ*g/mL for NPX, 0.5–3 *μ*g/mL CAF, and 1–15 *μ*g/mL for ASA and IPF. In addition, a validation set of an additional 15 samples containing the studied drugs were similarly prepared using different concentrations within the calibration range of each drug. Tables 1 and 2 show the calibration and validation sets, respectively. The spectra of all mixtures were recorded in the wavelength ranging from 220 to 240 nm with 0.8 nm interval. The data were exported into Matlab for developing the MCR-ALS model in which five variables were used for all the studied analytes.

### 2.5. Analysis of the Commercial Pharmaceutical Formulations

A real challenge of the presented work would be to quantify the studied drugs in commercial products presenting different compositions and interferences. In this study, ten commercial pharmaceutical tablets were collected from the local pharmacies (Eastern Province, Saudi Arabia) with highly variable compositions regarding the excipients and some containing other active ingredients such as codeine phosphate. The specified concentration level was described on the label of all the analyzed samples. The composition of the analyzed samples is as follows: (1) Fevadol Plus® tablets (SPIMACO) labeled to contain 500 mg of PAR, 30 mg of CAF, and 8 mg codeine per tablet, (2) Panadol Extra® tablets (SPIMACO) labeled to contain 500 mg of PAR and 65 mg of CAF per tablet, (3) Panadol® tablets (GSK) labeled to contain 500 mg of PAR per tablet, (4) Adol® tablets (Julphar) labeled to contain 500 mg of PAR per tablet, (5) Proxen® tablets (Grunenthal) labeled to contain 500 mg of NPX per tablet, (6) Omarfen® tablets (National Pharmaceutical Industries) labeled to contain 400 mg of IPF per tablet, (7) Disprin® tablets (Riyadh Pharma) labeled to contain 81 mg of ASA per tablet. (8) Fevadol® tablets (SPIMACO) labeled to contain 500 mg of PAR per tablet, (9) Riaproxe® tablets (Riyadh pharma) labeled to contain 500 mg of NPX per tablet, and (10) Brufen® tablets (Abbott) labeled to contain 500 mg of IPF per tablet.

Ten tablets of each pharmaceutical product were separately weighed and finely powdered. A portion of the powder equivalent to the average tablet weight of each product was separately dissolved in 50 mL methanol using ultrasonication for 15 min. Then, the solution was cooled and filtrated in a 100 mL volumetric flask using Whatman® filter papers. Finally, the volume was completed to 100 mL with methanol and suitable volumes of the stock solutions were mixed and diluted with ultrapure water to obtain different concentrations of the studied drugs at the specified linearity range mentioned above. The sample spectra were recorded using the same procedures described for the calibration and test sample sets.

### 2.6. Multivariate Calibration Analysis (MCR-ALS)

MCR-ALS is a chemometric method that provides relevant information about the pure components by decomposing the bilinear data matrix. In MCR-ALS, the matrix of mixed signals (*D*) is decomposed into concentration and signal profiles according to the following equation:

(1) $$\begin{array}{c} D=CS^{\text{T}}+E, \end{array}$$

where *C* is the concentration matrix, *S* is the signal matrix, and *E* is the residual matrix (the matrix associated to experimental error).

For optimizing the MCR-ALS model and achieving solutions for *C* and *S*, various constraints can be employed such as nonnegativity, closure, unimodality, and correlation constraints [22–25]. Nonnegativity constraint enforces the concentration and/or spectral matrices to have values equal to or greater than zero. Correlation constraint allows quantitative analysis of analytes in presence of unknown interfering components leading to enhanced accuracy of the analysis [26]. In this work, both nonnegativity and correlation constraints are used. For iteration procedures, initially spectral data matrix *D* is decomposed into bilinear data matrix according to Equation (1), followed by determination of the optimum number of variables for resolution. Then, *C* and *S*^T^ are initially estimated using simple-to-use interactive self-modeling mixture analysis (SIMPLISMA) [26]. Initial estimates of *C* and *S*^T^ is an important step because different estimates may lead to different results. Then, the developed calibration model is used for predicting concentrations in samples of both the validation and test sets. Each ALS iteration is completed after updating the obtained predicted values.

### 2.7. Validation of the Model

An external validation data set of 15 laboratory-prepared mixtures was used to evaluate the quantitative performance of the developed method by predicting the concentration of the studied drugs in a validation set which was not used for the development of the model (Table 2). The ability of the proposed model for predicting concentrations was assessed by calculating different figures of merit such as root mean square error of cross validation (RMSECV), root mean square error of prediction (RMSEP), bias, standard error of prediction (SEP), and relative percentage error in the concentration predictions (RE%) according to the following equations:

(2) $$\begin{array}{c} \text{RMSEP}=\;\;\sqrt{\frac{\sum_{i=1}^{n}{\left(c_{i}-{\hat{c}}_{i}\right)}^{2}}{n},}\\ \text{bias}=\frac{\sum_{i=1}^{n}\left(c_{i}-{\hat{c}}_{i}\right)}{n},\\ \text{SEP}=\sqrt{\frac{\sum_{i=1}^{n}{\left(c_{i}-{\hat{c}}_{i}-\text{bias}\right)}^{2}}{n-1}},\\ \text{RE }\left(\%\right)=\sqrt{\frac{\sum_{i=1}^{n}{\left(c_{i}-{\hat{c}}_{i}\right)}^{2}}{\sum_{i=1}^{n}c_{i}^{2}}}, \end{array}$$

where *c*_*i*_ and ${\hat{c}}_{i}\;\;$ are the known and predicted analyte concentrations in the sample *i*, respectively, and *n* is the total number of samples used in the validation set. Additionally, coefficients of determination (*r*^2^) were calculated for each analyte in the validation set.

The accuracy of the proposed chemometric method for the measurement of the studied drugs was tested by using the standard addition technique at 80%, 100%, and 120% of the test concentration. The study was performed by addition of known amounts of the studied drugs into a known concentration of the commercial pharmaceutical tablets. The resulting mixtures were analyzed, and the results obtained were compared with the expected results. The recovery of the exogenous amount added was calculated by the following equation:

(3) $$\begin{array}{c} \text{recovery}\left(\%\right)=\frac{\left(X-Y\right)}{ZZ}, \end{array}$$

where recovery means the drug recovered (%), *Y* expresses the normal concentration of sample before addition, *X* is the concentration of sample after addition of pure drug, and *Z* indicates the added amount.

Intraday precision (repeatability) and interday precision (reproducibility) of the developed method were evaluated by measuring three concentration levels that cover lower, middle, and upper limits of calibration curves.

## 3. Results and Discussion

### 3.1. Features of the Spectra

**Figure 1:**
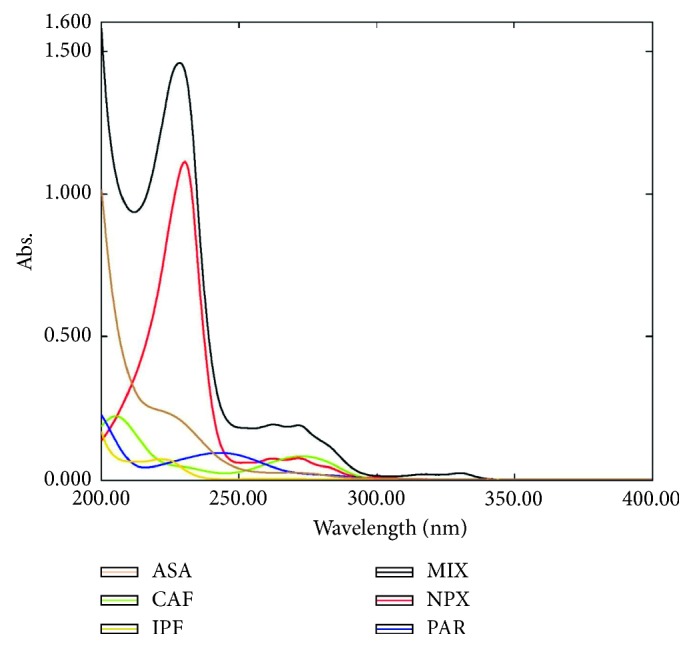
UV absorption spectra of 1 *μ*g/mL of paracetamol (PAR), aspirin (ASA), ibuprofen (IPF), naproxen (NPX), caffeine (CAF), and their mixture.

Figure 1 illustrates the UV spectra of the studied drugs PAR, ASA, CAF, NPX, IPF, and their mixture. As can be observed, the spectra are severely overlapped; therefore, determination of such drugs simultaneously in their mixtures cannot be accomplished utilizing univariate spectrophotometric methods because of the severe spectral overlap. As a result, the MCR-ALS method was proposed to improve the analysis of the studied drugs.

### 3.2. Selection of the Spectral Zones

The appropriate selection of the wavelength range is an important factor that strongly affects the quality of multivariate analysis [25]. Wavelengths lower than 220 nm were excluded because of the presence of noise in the spectra of the laboratory-prepared mixtures and pharmaceutical samples in this range. Additionally, wavelengths higher than 240 nm were rejected as well because of poor absorption of NPX and IPF at the measured concentration levels. Overall, absorbance data of the spectral region from 220 to 240 nm were found to be the optimum chemometric region for the quantitative determination of the studied drugs using the MCR-ALS model. After selecting the wavelength interval suitable for the analysis of the five drugs in their mixtures, commercial tablets were analyzed following the same procedures.

### 3.3. MCR-ALS Model

Optimization of the MCR-ALS model is a very important step in order to achieve optimum performance of the model. Because of the variability in ratios of the studied drugs, each drug was calibrated individually to provide good results. Proper selection of the latent variables (LVs) number is important to achieve correct quantitation. The optimum number of LVs should be the smallest number that results in no significant difference between RMSECV of that factor and the next one [27]. In this study, the optimum LVs number using the leave-one-out cross-validation technique was five for all analyzed drugs.

**Figure 2:**
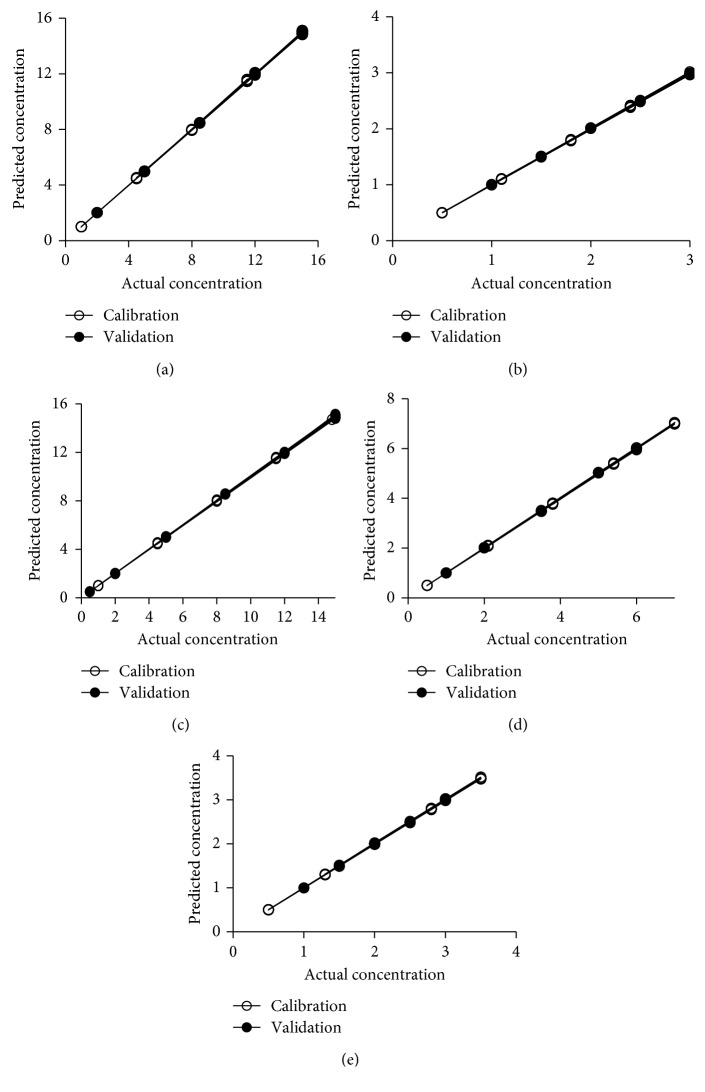
Plots of actual concentrations versus predicted values of the studied drugs for the calibration and validation sets using the MCR-ALS model. (a) IPF. (b) CAF. (c) ASA. (d) PAR. (e) NPX.

For MCR-ALS, different constraints were optimized and satisfactory results were obtained when applying nonnegativity constraints for spectral and concentration matrices, in addition to a correlation constraint. The convergence criterion was set at 0.1%, and the maximum number of iterations was 50; however, no more than 8 iterations were required to achieve convergence in all tested samples.  Table 3 shows the figures of merit of the calibration set of the model. The results show low relative error (RE) (%) ≤ 1.65 for all the studied drugs and low RMSECV (≤0.194). Coefficients of determination (*r*^2^) ≥ 0.9993 were achieved for all studied drugs which indicated the good ability of the model for prediction. The scatter plots of resolved MCR-ALS concentration values versus the actual concentrations for the calibration set are presented in Figure 2.

### 3.4. Method Validation

The developed model was validated by predicting the concentrations of the studied drugs in an external validation set (fifteen mixtures) which are not included in the model development. Different parameters such as RMSEP, SEP, RE (%), and *r*^2^ were calculated to assess the predictive ability of the developed model for the validation set (Table 4). The quality of the validation data is indicated by low relative errors (REs) ≤ 1.59% for all the studied drugs. The absorption spectra of each drug as well as their mixtures were checked for their linearity, thereby fulfilling the law of additivity for Beer's law (Figure 1). They were found to be linear in the ranges of 0.5–7 *μ*g/mL for PAR, 0.5–3.5 *μ*g/mL for NPX, 0.5–3 *μ*g/mL CAF, and 1–15 *μ*g/mL for ASA and IPF. As can be seen in the figure, the UV spectra of the mixture are equivalent to the sum of the pure spectra of the studied analytes reflecting the law of spectral additivity.

Satisfactory validation results were obtained and showed the high predictive ability of the model. The plots of the predicted concentrations versus the actual concentrations for the validation set are also illustrated in Figure 2. The high linearity (coefficient of determinations ≥ 0.9991) indicated the high prediction ability of the model.

The accuracy of the proposed method was evaluated by the standard addition method. The percent recoveries ranged from 98.0% to 99.7% with% RSDs not higher than 1.62% (Table 4). These results confirmed that the excipients in pharmaceutical products do not interfere with the determination of the studied drugs. Intraday precision and interday precision of the developed method were also examined by analyzing three concentration levels of the studied drugs within the same day for intraday precision and at three consecutive days for interday precision. The lower values of % RSD (≤1.34) indicated good precision of the developed method (Table 4).

### 3.5. Analysis of Commercial Pharmaceutical Products

The proposed chemometric method was used to analyze the studied drugs in commercial tablets. Five replicate determinations were performed.  Table 5 represents the results for the quantification of the studied drugs in their commercial tablets. The results of the proposed MCR-ALS model was statistically compared with a reported HPLC method [28] using paired *t*-test and *F*-ratio at 95% confidence level. There was no significant difference between the proposed and the reference method regarding accuracy and precision (Table 5).

## 4. Conclusion

An eco-friendly, fast, and accurate MCR-ALS chemometric method for the quantification of the most widely consumed OTC pain relievers has been developed and validated. The proposed model proved to be a green, nondestructive, and low-cost alternative to chromatographic techniques for the determination of the studied drugs in pure and commercial pharmaceutical formulations. The developed MCR-ALS method can be used as a green alternative for the analysis of the studied drugs without sample preparation steps and expensive solvents, especially in developing countries where resources are limited.

## Figures and Tables

**Table 1:** Concentration matrix (*μ*g/mL) used for the calibration set of the studied drugs.

Mixture	PAR	ASA	CAF	NPX	IPF
1	3.8	8.0	1.8	2.0	8.0
2	3.8	1.0	0.5	3.5	4.5
3	0.5	1.0	3.0	1.3	15.0
4	0.5	15.0	1.1	3.5	8.0
5	7.0	4.5	3.0	2.0	4.5
6	2.1	15.0	1.8	1.3	4.5
7	7.0	8.0	1.1	1.3	11.5
8	3.5	4.5	1.1	2.8	15.0
9	2.1	4.5	2.4	3.5	11.5
10	2.1	11.5	3.0	2.8	8.0
11	5.4	15.0	2.4	2.0	15.0
12	7.0	11.5	1.8	3.5	15.0
13	5.4	8.0	3.0	3.5	1.0
14	3.8	15.0	3.0	0.5	11.5
15	7.0	15.0	0.5	2.8	1.0
16	7.0	1.0	2.4	0.5	8.0
17	0.5	11.5	0.5	2.0	11.5
18	5.4	1.0	1.8	2.8	11.5
19	0.5	8.0	2.4	2.8	4.5
20	3.8	11.5	2.4	1.3	1.0
21	5.4	11.5	1.1	0.5	4.5
22	5.4	4.5	0.5	1.3	8.0
23	2.1	1.0	1.1	2.0	1.0
24	0.5	4.5	1.8	0.5	1.0
25	2.1	8.0	0.5	0.5	15.0

**Table 2:** Concentration matrix (*μ*g/mL) used for the validation set of the studied drugs.

Mixture	PAR	ASA	CAF	NPX	IPF
1	3.5	0.5	2.0	2.0	8.5
2	3.5	2.0	1.0	2.0	5.0
3	1.0	2.0	3.0	1.5	15.0
4	1.0	15.0	1.5	3.0	8.5
5	6.0	5.0	3.0	2.0	5.0
6	2.0	15.0	2.0	1.5	5.0
7	6.0	8.5	1.5	1.5	12.0
8	3.5	5.0	1.5	2.5	15.0
9	2.0	5.0	2.5	3.0	12.0
10	2.0	12.0	3.0	2.5	8.5
11	5.0	15.0	2.5	2.0	15.0
12	6.0	12.0	2.0	3.0	15.0
13	5.0	8.5	3.0	3.0	2.0
14	3.5	15.0	3.0	1.0	12.0
15	6.0	15.0	1.0	2.5	2.0

**Table 3:** Figures of merits of the calibration set for the developed MCR-ALS model.

Parameters	PAR	ASA	CAF	NPX	IPF
Calibration range	0.5–7	1–15	0.5–3	0.5–3.5	1–15
Slope	1.0000	0.9999	1.0000	1.0000	0.9999
Intercept	8.75 × 10^−15^	−1.96 × 10^−14^	−2.02 × 10^−13^	2.51 × 10^−3^	2.13 × 10^−3^
RMSECV	0.136	0.045	0.194	0.152	0.064
SEP	0.153	0.032	0.170	0.149	0.063
Bias	2.64 × 10^−2^	4.12 × 10^−3^	−2.58 × 10^−2^	3.22 × 10^−2^	3.11 × 10^−3^
RE (%)	1.32	0.94	1.65	1.28	1.15
Coefficient of determination (*r*^2^)	0.9998	0.9995	0.9993	0.9998	0.9996

**Table 4:** Validation parameters of the developed MCR-ALS model.

Parameter	PAR	ASA	CAF	NPX	IPF
Accuracy^a^	98.1 ± 1.28	99.5 ± 0.99	98.0 ± 1.12	98.9 ± 1.02	99.7 ± 1.62
Intraday precision^b^	0.93	0.85	1.23	1.02	1.23
Interday precision^c^	1.02	0.94	1.31	1.34	1.27
RMSEP	0.251	0.099	0.284	0.232	0.160
SEP	0.753	0.092	0.250	0.219	0.103
Bias	3.54 × 10^−2^	4.99 × 10^−3^	−3.98 × 10^−2^	4.29 × 10^−2^	4.11 × 10^−3^
RE (%)	1.52	1.59	1.35	1.49	1.43
Correlation coefficient (*r*^2^)	0.9993	0.9994	0.9991	0.9996	0.9992

^a^Mean ± standard deviation for 15 determinations. ^b^The intraday relative standard deviation (*n* = 3), average of three different concentrations repeated three times within the day. ^c^The interday relative standard deviation (*n* = 3), average of three different concentrations repeated three times in three different days.

**Table 5:** Determination of the studied drugs in commercial tablets by the MCR-ALS model by the proposed and the reported HPLC method.

		MCR-ALS	HPLC
Sample 1	PAR		
	(Mean + SD)	99.22 ± 1.11	99.86 ± 0.86
	*t*	0.86	—
	*F*	1.69	—
	CAF		
	(Mean + SD)	99.74 ± 0.72	99.8 ± 0.70
	*t*	0.50	—
	*F*	1.08	—

Sample 2	PAR		
	(Mean + SD)	99.06 ± 0.96	99.6 ± 0.83
	*t*	1.61	—
	*F*	1.35	—
	CAF		
	(Mean + SD)	99.14 ± 1.28	99.0 ± 1.44
	*t*	0.09	
	*F*	0.79	

Sample 3	PAR		
	(Mean + SD)	100.1 ± 1.11	100.5 ± 1.27
	*t*	0.80	—
	*F*	0.76	—

Sample 4	PAR		
	(Mean + SD)	99.5 ± 1.45	100.5 ± 1.47
	*t*	1.07	-
	*F*	0.97	-

Sample 5	NPX		
	(Mean + SD)	98.7 ± 1.50	99.9 ± 1.25
	*t*	2.67	—
	*F*	1.44	—

Sample 6	IPF		
	(Mean + SD)	99.6 ± 1.77	99.1 ± 1.49
	*t*	0.59	—
	*F*	1.40	—

Sample 7	ASA		
	(Mean + SD)	100.2 ± 1.85	99.9 ± 1.58
	*t*	0.34	—
	*F*	1.37	—

Sample 8	PAR		
	(Mean + SD)	100.82 ± 1.34	100.4 ± 1.45
	*t*	0.35	—
	*F*	0.85	—

Sample 9	NPX		
	(Mean + SD)	99.7 ± 1.55	100.4 ± 1.45
	*t*	0.93	—
	*F*	1.14	—

Sample 10	IPF		
	(Mean + SD)	100.2 ± 1.68	99.5 ± 1.54
	*t*	0.56	—
	*F*	1.19	—

The reference HPLC published method used C_18_ (250 × 4.6 mm, 5.0 *μ*m) column at 35°C, and the mobile phase composed of 15 mM phosphate buffer (pH 3.25) and acetonitrile using gradient elution at 1.1 mL·min^−1^ flow rate. SD: standard deviation of the mean of the percentage recovery from the label claim amount for 5 determinations. Theoretical values for *t* and *F* at *p*=0.05 are 2.78 and 6.39, respectively.

## Data Availability

The data used to support the findings of this study are available from the corresponding author upon request.
